# Muscleblind-like 3 deficit results in a spectrum of age-associated pathologies observed in myotonic dystrophy

**DOI:** 10.1038/srep30999

**Published:** 2016-08-03

**Authors:** Jongkyu Choi, Donald M. Dixon, Warunee Dansithong, Walid F. Abdallah, Kenneth P. Roos, Maria C. Jordan, Brandon Trac, Han Shin Lee, Lucio Comai, Sita Reddy

**Affiliations:** 1 Department of Biochemistry and Molecular Biology, University of Southern California, Los Angeles, CA 90033, USA; 2 USC Eye Institute, Los Angeles, CA 90033, USA; 3 Department of Ophthalmology, Faculty of Medicine, Zagazig University, Zagazig, Egypt; 4 Department of Physiology, David Geffen School of Medicine at UCLA, Los Angeles, CA 90095-1751, USA; 5 Department of Microbiology and Immunology, University of Southern California, Los Angeles, CA 90033, USA

## Abstract

Myotonic dystrophy type I (DM1) exhibits distinctive disease specific phenotypes and the accelerated onset of a spectrum of age-associated pathologies. In DM1, dominant effects of expanded *CUG* repeats result in part from the inactivation of the muscleblind-like (MBNL) proteins. To test the role of MBNL3, we deleted Mbnl3 exon 2 (*Mbnl3*^*ΔE2*^) in mice and examined the onset of age-associated diseases over 4 to 13 months of age. Accelerated onset of glucose intolerance with elevated insulin levels, cardiac systole deficits, left ventricle hypertrophy, a predictor of a later onset of heart failure and the development of subcapsular and cortical cataracts is observed in *Mbnl3*^*ΔE2*^ mice. Retention of embryonic splice isoforms in adult organs, a prominent defect in DM1, is not observed in multiple RNAs including the Insulin Receptor (*Insr*), Cardiac Troponin T (*Tnnt2*), Lim Domain Binding 3 (*Ldb3*) RNAs in *Mbnl3*^*ΔE2*^ mice. Although rare DM1-like splice errors underlying the observed phenotypes cannot be excluded, our data in conjunction with the reported absence of alternative splice errors in embryonic muscles of a similar *Mbnl3*^*ΔE2*^ mouse by RNA-seq studies, suggest that mechanisms distinct from the adult retention of embryonic splice patterns may make important contributions to the onset of age-associated pathologies in DM1.

Myotonic Dystrophy type 1 (DM1) is a multi-system disorder resulting from the expansion of a CTG repeat sequence located in the 3′ untranslated region of *DMPK* and immediately 5′ of *SIX5*^1^,^2^,^3^. DM1 manifests with both age-associated pathologies and unique disease specific features that occur with varying severity and incidence. This array of features include skeletal muscle myotonia and weakness, a complex cardiac pathology that manifests as arrhythmias, systole dysfunction, ventricular hypertrophy and heart failure, abnormal glucose metabolism, elevated insulin levels, formation of ocular cataracts that are primarily sub-capsular and cortical in origin, testicular atrophy, frontal balding, hypersomnia and the development of cognitive and emotional deficits^1^,^4^,^5^.

Multiple lines of evidence demonstrate that dominant RNA effects resulting from the expression of mutant *DMPK* RNAs encoding expanded *CUG* repeat sequences (*CUGexp*) play a central role in the development of key aspects of DM1 pathology^6^,^7^,^8^,^9^. In other studies, we and others have shown that reductions in DMPK and SIX5 levels, which occur as a consequence of nuclear aggregation of the mutant *DMPK* RNA encoding *CUGexp* with the MBNL family of proteins and allele specific silencing of *SIX5* expression resulting from CTG tract expansion respectively, are sufficient to result in a subset of DM1 cardiac, skeletal muscle, ocular and gonad pathologies^10^,^11^,^12^,^13^,^14^,^15^,^16^,^17^,^18^,^19^,^20^.

RNA dominant effects in DM1 have been hypothesized to stem in part from the ability of *CUGexp* to aberrantly sequester and functionally inactivate members of the muscleblind (MBNL) family of proteins^10^,^11^,^12^, increase steady-state levels of CUG-BP1 and other RNA binding proteins and from the nuclear exclusion of SHARP^8^,^21^,^22^. Consistent with this hypothesis, previous studies have demonstrated that Mbnl1 depletion in mice results in skeletal muscle myotonia, dust-like ocular cataracts, cardiac arrhythmias and both behavioral and motivational deficits^23^,^24^,^25^. In other studies, Mbnl2 loss has been shown to increase seizure susceptibility, induce REM sleep abnormalities and deficits in spatial memory^26^. Concordantly, a wide array of RNA splice errors has been documented in *Mbnl1*^*Δ3/Δ3*^ muscle, heart and brain and in *Mbnl2*^*Δ2/Δ2*^ brain^26^,^27^. In mirror image experiments over expression of CUG-BP1 has been shown to result in muscle dysfunction, cardiac arrhythmias and dilated cardiomyopathy^28^,^29^. There is a significant overlap in the splice defects that result from MBNL loss and CUG-BP1 overexpression^30^. Thus altered splicing of RNAs targeted by the MBNL protein family and CUG-BP1 has been hypothesized to result in DM1 pathology.

Experiments directed at the examination of the causal role of RNA splice errors in the development of DM1 features are limited to myotonia, where reversion of *Clc-1* RNA splice defects has been shown to rescue myotonia in the *HSA*^*LR*^ DM1 mouse model^31^. Aberrant splicing of the insulin receptor (*Insr*) RNA has been implicated in the development of abnormal glucose tolerance in DM1^32^. These initial studies and other related experiments have led myotonic dystrophy to be viewed as an RNA spliceopathy. The requirement of the characteristic splice errors observed in DM1, which largely manifest as the retention of embryonic RNA splice patterns in adult tissues^26^,^27^,^30^,^33^, as the primary triggering mechanism responsible for the manifestation of all aspects of DM1 pathophysiology, is however currently unclear.

In this study we tested the role of the third member of the muscleblind family, MBNL3, in DM1 etiology. We deleted exon 2 of *Mbnl3*, which is an X-linked gene, to develop *Mbnl3*^*ΔE2*^ mice^34^. This mutation results in the absence of the full-length 38 kD Mbnl3 protein (Mbnl3_38kD_) and the retention of a truncated 27 kD Mbnl3 isoform (Mbnl3_27kD_) translated from an ATG codon present in *Mbnl3* exon 3, as previously described by us and Poulos and colleagues^34^,^35^. *Mbnl3*^*ΔE2*^ mice demonstrate an accelerated onset of a subset of age-associated phenotypes observed in DM1, over a range of 4–13 months of age. Specifically, Mbnl3_38kD_ deficits trigger the early onset of abnormal glucose metabolism, elevated insulin levels, cardiac systole dysfunction that progresses to left ventricle hypertrophy, and a high incidence of subcapsular and cortical cataract formation. To test if DM1 specific splice errors contribute to the development of these phenotypes, we studied the splice patterns of twenty RNAs including the Insulin Receptor (*Insr*), Lim Binding Domain 3 (*Ldb3*) and Cardiac Troponin T (*Tnnt2*) RNAs in *Mbnl3*^*ΔE2*^ mice. DM1-like splice errors are not observed in these RNAs and reversion to the embryonic splice patterns is not observed for the *Insr* and *Tnnt2* RNAs in *Mbnl3*^*ΔE2*^ skeletal muscle and heart. The modest splice error detected in the *Ldb3* RNA in *Mbnl3*^*ΔE2*^ hearts does not recapitulate the embryonic splice pattern. Thus these data demonstrate Mbnl3_38kD_ deficits can cause the accelerated onset of age-associated DM1 pathologies and suggest that mechanisms distinct from splice alterations may contribute to the development of such DM1 phenotypes.

## Results

### Abnormal glucose tolerance in *Mbnl3* ^*ΔE2*^ mice at 7–9 months of age

To test the potential effects of MBNL3 deficiency in the development of DM1 pathophysiology we developed *Mbnl3*^*ΔE2*^ mice on a 129sv background, in which exon 2 of the X-linked *Mbnl3* gene was replaced by a Neomycin expression cassette^34^. As previously reported^12^,^34^ we observe Mbnl3 expression in the adult mouse spleen and diminished but clearly detectable *Mbnl3* mRNA expression in the adult soleus muscle and in the adult mouse heart, lens and brain^34^ (Supplementary Figure S1).

**Figure 1:**
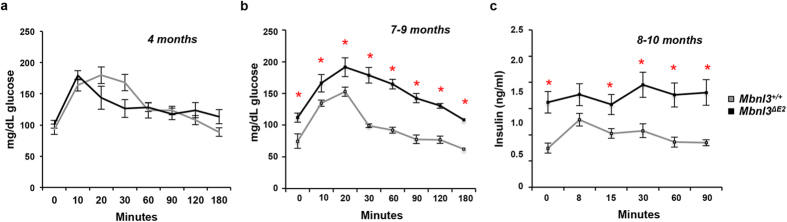
*Mbnl3*^*ΔE2*^ mice demonstrate abnormal glucose metabolism and elevated insulin levels at 7–10 months of age. (**a,b**) Blood glucose levels at the time points indicated subsequent to dextrose injection at time zero for male *Mbnl3*^+/+^ and male *Mbnl3*^*ΔE2*^ mice at 4 months (*Mbnl3*^+/+^ n = 4 and *Mbnl3*^*ΔE2*^ n = 3; Panel a) and 7–9 months of age (*Mbnl3*^+/+^ n = 5 and *Mbnl3*^*ΔE2*^ n = 7; Panel b) are shown. At 7–9 months of age, there was a significant difference in mean blood glucose levels between groups (*Mbnl3*^+/+^ and *Mbnl3*^*ΔE2*^ mice) adjusting for reads and time (mean difference 51.1358; 95% CI = 28.4037, 73.8679; p < 0.0001). (**c**) Blood insulin levels at the time points indicated subsequent to dextrose injection at time zero for male *Mbnl3*^+/+^ and male *Mbnl3*^*ΔE2*^ mice at 8–10 months (*Mbnl3*^+/+^ n = 4 and *Mbnl3*^*ΔE2*^ n = 5) are shown. The incidence rate ratio (IRR) is significantly different between groups (*Mbnl3*^+/+^ and *Mbnl3*^*ΔE2*^ mice) adjusting for reads and time (IRR = 1.798; 95% CI = 1.1202, 2.8854; p = 0.015). p-values were calculated using the Student’s t-test with significance set at p ≤ 0.05. Values where p ≤ 0.05 are denoted by a red asterisk. Data are standard error of mean.

Glucose metabolism was assessed at 4 and 7–9 months of age in male *Mbnl3*^+/+^ and *Mbnl3*^*ΔE2*^ mice. Briefly, mice were fasted for 6 hours prior to testing and baseline blood sugar levels were obtained from a drop of tail blood. Subsequently, a bolus of sterile 5% dextrose in saline was injected IP at a dose of 1 g/kg at time zero and blood glucose levels were repeatedly tested for up to 3 hours following the injection. Area under the curve for the glucose tolerance test was calculated using an individual t-test at 4 months of age for male *Mbnl3*^*ΔE2*^ (n = 3) and *Mbnl3*^+/+^ (n = 4) mice. At 4 months of age, there was no statistically significant difference in blood glucose levels (t_5_ = 0.163, p = 0.88). However, a trend towards a shorter time for peak glucose levels to be achieved and elevated blood glucose levels 3 hours post injection was observed in *Mbnl3*^*ΔE2*^ mice when compared to *Mbnl3*^+/+^ controls (Fig. 1a; Supplementary Table S1a).

At 7–9 months of age, baseline blood glucose levels were significantly different between male *Mbnl3*^+/+^ (n = 5) and *Mbnl3*^*ΔE2*^ mice (n = 7) (p < 0.001). Upon dextrose injection, peak glucose levels achieved were both significantly higher and were sustained at higher levels for a longer duration, with blood glucose levels ~57% higher in *Mbnl3*^*ΔE2*^ mice when compared to control animals, 3 hours post injection (p < 0.013) (Fig. 1b; Supplementary Table S1b). At 7–9 months of age, there was a significant difference in mean blood glucose levels between groups (*Mbnl3*^+/+^ and *Mbnl3*^*ΔE2*^ mice) adjusting for reads and time (mean difference 51.1358; 95% CI = 28.4037, 73.8679; p < 0.0001).

### *Mbnl3* ^*ΔE2*^ mice have elevated insulin levels

To test if elevated blood glucose levels subsequent to dextrose injection in *Mbnl3*^*ΔE2*^ mice was due to decreased insulin levels, we measured insulin levels in 8–10 month old *Mbnl3*^+/+^ and *Mbnl3*^*ΔE2*^ mice, at 0, 8, 15, 30 and 60 minutes following the intraperitonel injection of dextrose at a dose of 2 g/kg, subsequent to a 16 hour fast as previously described^36^. Blood insulin levels were significantly higher in *Mbnl3*^*ΔE2*^ mice when compared to *Mbnl3*^+/+^ mice prior to dextrose injection and the incidence rate ratio (IRR) is significantly different between groups (*Mbnl3*^+/+^ and *Mbnl3*^*ΔE2*^ mice) adjusting for reads and time (IRR = 1.798; 95% CI = 1.1202, 2.8854; p = 0.015), demonstrating an 80% likelihood of higher insulin levels in the *Mbnl3*^*ΔE2*^ mouse group when compared to control mouse group (Fig. 1c; Supplementary Table S2).

### *Insr* exon 10a splicing is normal in *Mbnl3* ^*ΔE2*^ skeletal muscle

**Figure 2:**
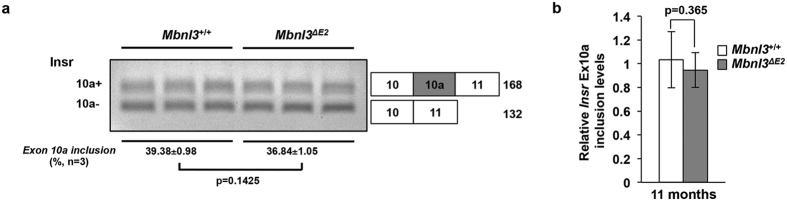
*Mbnl3*^*ΔE2*^ muscles do not show altered *Insr* exon 10a splicing. (**a**) *Insr* exon 10a inclusion is not significantly different in male *Mbnl3*^*ΔE2*^ soleus muscles when compared to male *Mbnl3*^+/+^ soleus muscles at 11 months of age. Data are standard error of mean (n = 3 soleus muscles from independent mice for each genotype). Exon numbers are annotated based on Refseq from UCSC genome browser (NCBI37/mm9). (**b**) Relative *Insr* exon 10a inclusion was quantitated by qPCR analysis using exon-exon boundary spanning primers specific to exon 10a (exon 10a–11) and to a constitutive exon (exon 9–10) at 11 months of age. Expression levels of *Insr* exon 10a were normalized to that of *Insr* exon 9. Data are standard error of mean (n = 3 soleus muscles from independent mice for each genotype; each sample was repeated in triplicate).

As aberrant splicing of exon 10a of the *Insr* RNA has been implicated in DM1-associated insulin resistance^32^ we tested *Insr* splicing in soleus muscles from 7 and 11 month male *Mbnl3*^+/+^ and *Mbnl3*^*ΔE2*^ mice. Significant differences were not observed for this splice event for the two genotypes using semi-quantitative RT-PCR reactions at 7 months (Supplementary Fig. S2) and 11 months (Fig. 2a) or by qPCR measurements at 11 months (Fig. 2b; qPCR: relative *Insr* exon 10a inclusion: *Mbnl3*^+/+^: 1.034 ± 0.079 and *Mbnl3*^*ΔE2*^: 0.947 ± 0.049; p = 0.365).

### *Mbnl3* ^*ΔE2*^ hearts show cardiac systole defects at 4 months and ventricular hypertrophy at 11 months of age

**Figure 3:**
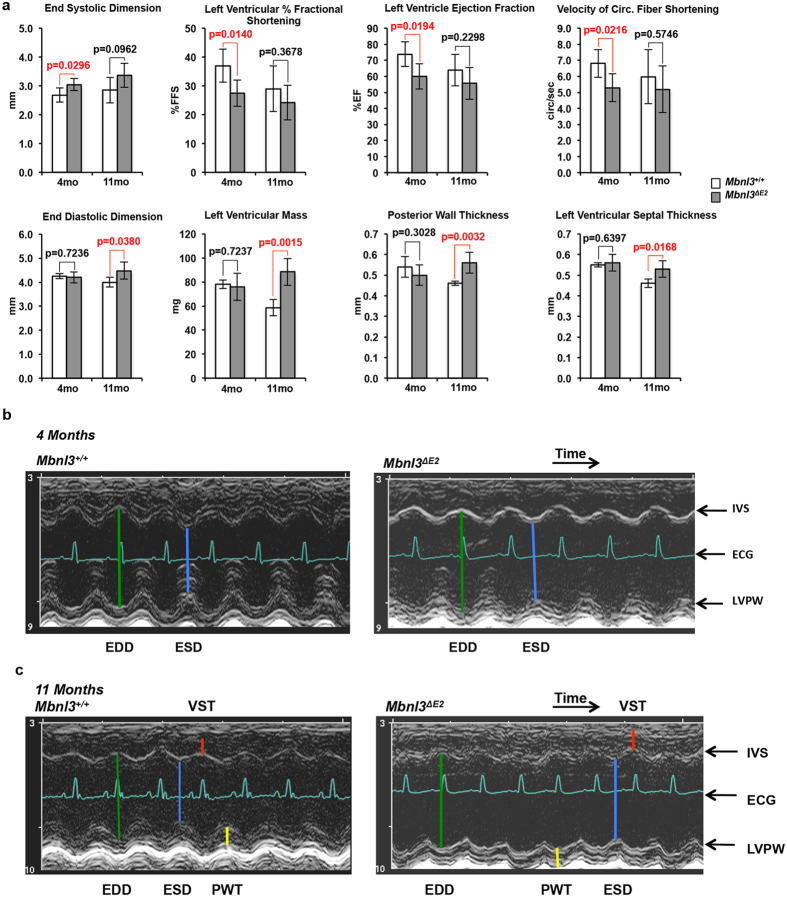
*Mbnl3*^*ΔE2*^ mice show systole dysfunction and ventricular hypertrophy. (**a**) Ultrasound echocardiographic evaluation of male *Mbnl3*^+/+^ and male *Mbnl3*^*ΔE2*^ mice at 4 months (*Mbnl3*^+/+^ n = 4 and *Mbnl3*^*ΔE2*^ n = 7) and 11 months of age (*Mbnl3*^+/+^ n = 4 and *Mbnl3*^*ΔE2*^ n = 6) is shown. Data are shown as mean and standard deviation. p-values were calculated using Student’s t-test with significance set at p ≤ 0.05. Values where p ≤ 0.05 are shown in red. (**b,c**) 2-D guided M-Mode images of 4 and 11 month old male *Mbnl3*^+/+^ and male *Mbnl3*^*ΔE2*^ hearts are shown. The depth from top to bottom is 7 mm. Time is 100 ms/div. Abbreviations: EDD: End-diastolic dimension (green). ESD: End-systolic dimension (blue). VST: Left ventricular septal thickness (red); PWT: Posterior wall thickness (yellow); IVS: interventricular septum; LVPW: left ventricular posterior wall; ECG: electrocardiogram.

Structure-function evaluation of the heart was carried out in male *Mbnl3*^+/+^ and *Mbnl3*^*ΔE2*^ mice as a function of age. Left ventricular function and chamber dimensions were evaluated in male *Mbnl3*^+/+^ and *Mbnl3*^*ΔE2*^ mice by ultrasound echocardiography at 4 months (*Mbnl3*^+/+^ n = 4, *Mbnl3*^*ΔE2*^ n = 7) and 11 months (*Mbnl3*^+/+^ n = 4, *Mbnl3*^*ΔE2*^ n = 6) of age (Fig. 3; Supplementary Table S3). These experiments demonstrate that at 4 months *Mbnl3*^*ΔE2*^ mice exhibit left ventricular systole dysfunction showing a significant increase in end-systolic dimension (*Mbnl3*^+/+^: 2.68 ± 0.25 mm and *Mbnl3*^*ΔE2*^: 3.04 ± 0.21 mm; p = 0.03) and a consequent reduction in left ventricular function manifesting as diminished left ventricle percent fractional shortening (*Mbnl3*^+/+^: 37.0 ± 5.7 and *Mbnl3*^*ΔE2*^: 27.5 ± 4.6; p = 0.01), left ventricle ejection fraction (*Mbnl3*^+/+^: 73.8 ± 7.7 and *Mbnl3*^*ΔE2*^: 59.9 ± 7.8; p = 0.02) and velocity of circumferential fiber shortening (*Mbnl3*^+/+^: 6.80 ± 0.86 and *Mbnl3*^*ΔE2*^: 5.29 ± 0.87; p = 0.02) when compared to *Mbnl3*^+/+^ animals.

Left ventricle systole dysfunction in male *Mbnl3*^*ΔE2*^ mice at four months of age occurs without differences in diastole chamber dimensions. In contrast, at 11 months of age, male *Mbnl3*^*ΔE2*^ mice exhibit a hypertrophic response, most likely in response to the cardiac systole deficits that manifest at 4 months of age. Specifically, significant increases in end-diastolic dimension (*Mbnl3*^+/+^: 4.00 ± 0.2 and *Mbnl3*^*ΔE2*^: 4.48 ± 0.35; p = 0.04), left ventricular mass (*Mbnl3*^+/+^: 58.7 ± 6.9 and *Mbnl3*^*ΔE2*^: 88.5 ± 11.2; p = 0.001), posterior wall thickness (*Mbnl3*^+/+^: 0.46 ± 0.01 mm and *Mbnl3*^*ΔE2*^: 0.56 ± 0.05 mm; p = 0.003) and ventricular septal thickness (*Mbnl3*^+/+^: 0.46 ± 0.02 mm and *Mbnl3*^*ΔE2*^: 0.53 ± 0.04 mm; p = 0.02) are observed in this age group. Finally, the E/A ventricular filling ratios are in the normal range suggesting no diastole dysfunction accompanying the hypertrophy. These age-related functional and morphological changes are shown in Fig. 3 and Supplementary Table S3.

As each sample set at 4 and 11 months of age are separate, typical corrections for multiple comparisons do not directly apply to these data. Given the small sample size, a statistically significant effect indicates very large effect sizes (Cohen’s Δ = 1.7, 2.3 for α = 0.05 and 0.01 respectively). Even with multiple comparison corrections, the most relevant outcomes of left ventricle percent fractional shortening at 4 months and left ventricular mass and posterior wall thickness at 11 months are significant.

Surface electrocardiograms were recorded in parallel from male *Mbnl3*^+/+^ and *Mbnl3*^*ΔE2*^ mice at 4 and 11 months of age. The wave shapes and intervals were within the normal range for *Mbnl3*^*ΔE2*^ mice at these time points (Supplementary Table S4). No arrhythmias or other abnormalities were noted. Histological analyses indicated normal structure with no pathology in both groups of *Mbnl3*^*ΔE2*^ mice (data not shown).

### *Mbnl3* ^*ΔE2*^ hearts demonstrate a minor splice defect in cardiac *Ldb3* RNA

**Figure 4:**
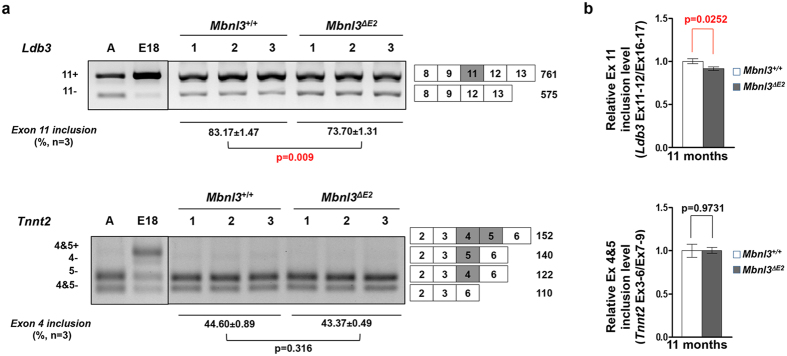
*Ldb3* is aberrantly spliced in *Mbnl3*^*ΔE2*^ hearts. (**a**) Alternative splicing was analyzed for *Ldb3* and *Tnnt2* in 11 months male *Mbnl3*^+/+^ (n = 3) and 11 month male *Mbnl3*^*ΔE2*^ (n = 3) hearts by RT-PCR. Band intensities were quantified by densitometry. *Ldb3* exon 11 inclusion in *Mbnl3*^*ΔE2*^ was significantly decreased when compared to *Mbnl3*^+/+^ hearts. *Tnnt2* exon 4 + 5 inclusion was not significantly different in *Mbnl3*^+/+^ and *Mbnl3*^*ΔE2*^ hearts. Exon numbers and expected band sizes are indicated. The alternatively spliced exon is shown as a gray box. Exon numbers are annotated based on Refseq from UCSC genome browser (NCBI37/mm9). Data are standard error of mean. (**b**) Expression levels of the alternatively spliced and the constitutive exons of *Ldb3* and*Tnnt2* were quantitated by qPCR using exon-exon boundary spanning primers specific to *Ldb3* exon 11 (exons 11–12), and *Tnnt2* exon 4 + 5 (exon 3–6) and the constitutive exons (exon 16–17 for *Ldb3*; exon 7–9 for *Tnnt2*). Expression levels of each alternatively spliced exon was normalized to that of the corresponding constitutive exon. Relative *Ldb3* exon 11 inclusion in *Mbnl3*^*ΔE2*^ hearts is significantly decreased when compared to *Mbnl3*^+/+^ hearts. Relative *Tnnt2* exon 4 + 5 inclusion is not significantly different in *Mbnl3*^+/+^ and *Mbnl3*^*ΔE2*^ hearts. Data are standard error of mean (n = 3 mice for each genotype, each sample was repeated in triplicate).

*Ldb3* and *Tnnt2* mutations are known to cause cardiac dysfunction, dilated cardiomyopathy and cardiac hypertrophy^37^,^38^. Significantly, re-expression of the embryonic *Tnnt2* isoforms in the adult heart is strongly associated with heart failure in humans^39^ and elevated expression of the embryonic isoform of *Tnnt2* in transgenic mice results in diminished cardiac efficiency^40^. We therefore tested if retention of embryonic splice isoforms of *Ldb3* and *Tnnt2* in adult hearts, two splice errors previously described in DM1^33^,^41^, are observed in 7 and 11 month old male *Mbnl3*^*ΔE2*^ hearts. *Ldb3* shows a modest but significant decrease in the inclusion of exon 11 in *Mbnl3*^*ΔE2*^ hearts when compared to *Mbnl3*^+/+^ hearts by RT-PCR at 7 months (Supplementary Fig. S2) and by RT-PCR and qPCR at 11 months (Fig. 4; RT-PCR *Ldb3* exon 11 inclusion: *Mbnl3*^+/+^: 83.17 ± 1.47% and *Mbnl3*^*ΔE2*^: 73.7 ± 1.31%; p = 0.009; qPCR: relative *Ldb3* exon 11 inclusion: *Mbnl3*^+/+^: 1.00 ± 0.028 and *Mbnl3*^*ΔE2*^: 0.913 ± 0.021; p = 0.025). This splice error is not reminiscent of the embryonic *Ldb3* exon 11 splicing pattern (Fig. 4a). The splice pattern of *Tnnt2* exon 4 and 5 is not altered in 7 or 11 month old *Mbnl3*^*ΔE2*^ hearts when compared to controls when examined by RT-PCR or qPCR (Fig. 4b; Supplementary Fig. S2; qPCR: relative *Tnnt2* exon 4 + 5 inclusion: *Mbnl3*^+/+^: 1.00 ± 0.076 and *Mbnl3*^*ΔE2*^: 1.003 ± 0.039; p = 0.973). Consistent with the results from the skeletal muscle, *Insr* and *Mbnl1* splice patterns are also not altered in *Mbnl3*^*ΔE2*^ hearts at either age (Supplementary Fig. S2; qPCR: relative *Insr* exon 10a inclusion: *Mbnl3*^+/+^: 0.997 ± 0.03 and *Mbnl3*^*ΔE2*^: 0.966 ± 0.023; p = 0.44).

To more generally test the possible incidence of DM1 like splice errors in *Mbnl3*^*ΔE2*^ hearts, we analyzed splice events in *Pdlim3/Alp, Trim55/Murf2, Mapt/Tau, Pdlim5, Sorbs1, Sorbs2, Fhod1, Spag9, Mbnl2, Myom1, Clta, Stx2, Csda, Sirt2, Atp2a1 and Atp11a* RNAs, which show the aberrant retention of embryonic splice isoforms in adult *Mbnl1*^*ΔE2/ΔE2*^ hearts^25^, in 11 month old *Mbnl3*^+/+^ and *Mbnl3*^*ΔE2*^ hearts and from E18 *Mbnl3*^+/+^ hearts. In 11 month *Mbnl3*^*ΔE2*^ hearts, none of splice events examined showed significant alterations when compared to 11 month old *Mbnl3*^+/+^ hearts (Supplementary Fig. S3)

In parallel these splice events were examined in 11 month old *Mbnl3*^+/+^ and *Mbnl3*^*ΔE2*^ soleus muscles and in E18 *Mbnl3*^+/+^ skeletal muscles. As with the *Mbnl3*^*ΔE2*^ heart, splice errors were not observed in 11 month old *Mbnl3*^*ΔE2*^ soleus muscles (Supplementary Fig. S4).

### *Mbnl3* ^*ΔE2*^ mice develop posterior subcapsular and cortical cataracts at 12 months of age

**Figure 5:**
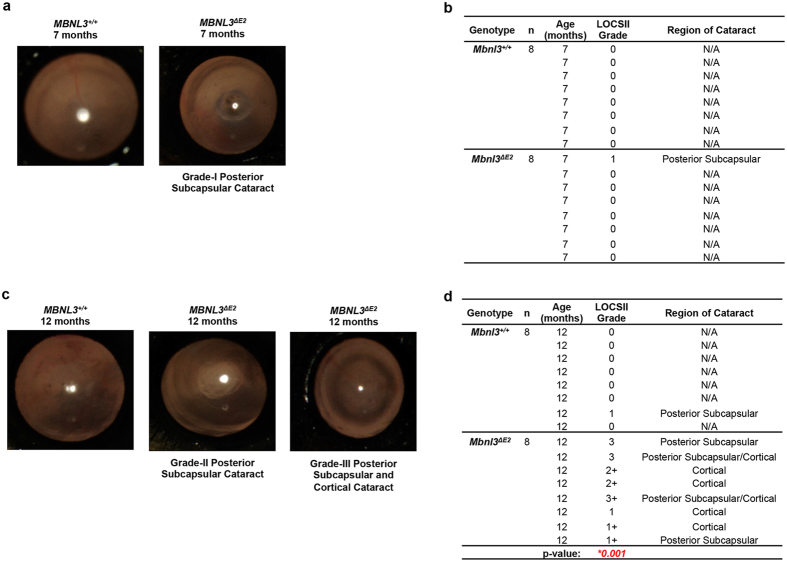
*Mbnl3*^*ΔE2*^ mice demonstrate subcapsular and cortical cataracts. (**a,c**) Fundus camera photographs of representative male *Mbnl3*^+/+^ and male *Mbnl3*^*ΔE2*^ mice at 7 months and 12 months of age respectively are shown. (**b,d**) Grade and location of cataracts in *Mbnl3*^+/+^ and *Mbnl3*^*ΔE2*^ mice at 7 months and 12 months are tabulated. Lenses were observed using a Haag Streit 900 slit lamp and cataracts were evaluated using the LOCS II photographic grading standards. Mann-Whitney U test demonstrates a statistically significant difference in cataract grade between *Mbnl3*^+/+^ and *Mbnl3*^*ΔE2*^ mice at 12 months of age (p = 0.001).

At 7 months of age, examination of *Mbnl3*^+/+^ (n = 8) and *Mbnl3*^*ΔE2*^ (n = 8) lenses, demonstrated a single Grade-I subcapsular cataract in *Mbnl3*^*ΔE2*^ mice. No *Mbnl3*^+/+^ lenses studied at this age showed cataract formation (Fig. 5a,b). At 12 months, Grade-I posterior subcapsular cataracts were detected in 1 of 8 *Mbnl3*^+/+^ lenses examined. In contrast, at 12 months of age, 100% of the *Mbnl3*^*ΔE2*^ lenses studied demonstrated cataract formation (n = 8), with ~60% showing advanced Grade-II and Grade-III cataracts as evaluated using LOCS II photographic grading standards. Cataracts in *Mbnl3*^*ΔE2*^ mice were primarily posterior subcapsular or cortical in origin (Fig. 5c,d). We performed the Mann-Whitney U test to compare cataract grades between *Mbnl3*^+/+^ and *Mbnl3*^*ΔE2*^ lenses at 12 months of age. This test demonstrates a statistically significant difference in cataract grade between *Mbnl3*^+/+^ and *Mbnl3*^*ΔE2*^ mice at 12 months of age (p = 0.001). Electroretinography studies did not show significant retinal defects in *Mbnl3*^*ΔE2*^ mice at 13 months of age (Supplementary Table S5). We examined splice events in *Mapt/Tau, Pdlim5, Sorbs1, Sorbs2, Spag9, Mbnl2, Stx2, Csda, Srt2, and Atp11a* RNAs in whole lenses from 8 month old *Mbnl3*^+/+^ and *Mbnl3*^*ΔE2*^ mice and from E18 *Mbnl3*^+/+^ mice. The splice events tested were not significantly different in adult *Mbnl3*^*ΔE2*^ and *Mbnl3*^+/+^ lenses (Supplementary Fig. S5).

## Discussion

**Figure 6:**
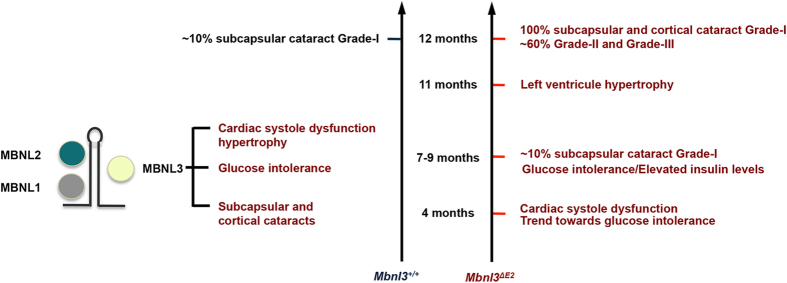
*Mbnl3*^*ΔE2*^ mice show an accelerated onset of DM1 age associated pathologies. Rate of onset of senescent features measured in this study for *Mbnl3*^+/+^ and *Mbnl3*^*ΔE2*^ mice are summarized.

Myotonic dystrophy type I (DM1) is a multi-system disorder, which exhibits the accelerated onset of several age-associated disease phenotypes that include abnormal glucose metabolism, left ventricle systolic deficits, hypertrophy and heart failure, ocular cataracts that originate in the sub-capsular and cortical regions of the lens^1^,^4^,^5^. DM1 patients also exhibit a variety of distinctive pathologies that do not generally manifest in normal human populations with aging such as skeletal muscle myotonia^1^. Several lines of evidence demonstrate that functional inactivation of the muscleblind (MBNL) family of proteins and an increase in CUG-BP1 play an important role in the development of DM1 pathology^23^,^24^,^25^,^26^,^28^,^29^. As both a decrease in MBNL1 and MBNL2 and an increase in CUG-BP1 levels are shown to facilitate retention of embryonic or neonatal splice isoforms in adult organs and reversion of DM1 splice defects in the chloride channel RNA is sufficient to rescue myotonia in mouse models, DM1 is widely recognized as an RNA spliceopathy^26^,^27^,^28^,^29^,^30^,^31^,^32^,^33^. In this study we demonstrate that deficits in Mbnl3_38kD_ result in a striking acceleration in the rate of onset of a set of DM1 age-associated pathologies (Fig. 6). Significantly, the accelerated onset of glucose intolerance with elevated insulin levels, cardiac systole deficits, hypertrophy, and ocular cataracts in *Mbnl3*^*ΔE2*^ mice occur largely without DM1-like splice defects in multiple RNAs. Splice errors in the *Insr* RNA, which have previously been implicated in the development of glucose intolerance in DM1^32^, are not observed in *Mbnl3*^*ΔE2*^ skeletal muscles. Other characteristic DM1 RNA splice defects in *Tnnt2* and *Ldb3* RNAs^33^,^41^, which are associated with cardiac dysfunction, are also not detected in *Mbnl3*^*ΔE2*^ cardiac muscles. Thus our results demonstrate that Mbnl3_38kD_ depletion can contribute to the development of age-associated pathologies observed in DM1. These experiments suggest that mechanisms distinct from the adult retention of embryonic splice isoforms may contribute to the onset of such age-associated phenotypes in DM1.

Endocrine abnormalities occur frequently in DM1, with patients having an increased risk of developing diabetes^1^,^42^,^43^. The majority of DM1 patients however rarely show overt diabetes, but can exhibit insulin resistance and glucose intolerance^1^,^43^. In our study we measured blood glucose levels subsequent to dextrose injection at 4 and 7–9 months of age in *Mbnl3*^*ΔE2*^ and *Mbnl3*^+/+^ mice. A trend towards elevated glucose levels was observed in *Mbnl3*^*ΔE2*^ mice at 4 months. At 7–9 months of age *Mbnl3*^*ΔE2*^ mice show aberrant glucose tolerance, with both higher peak glucose levels and sustained elevated glucose levels that did not return to baseline 180 minutes post injection. Overt diabetes was not observed at either age and baseline blood glucose levels were not significantly elevated in *Mbnl3*^*ΔE2*^ mice when compared to *Mbnl3*^+/+^ mice. In DM1 patients an increase in basal blood insulin levels is reported^44^,^45^. We observe elevated basal blood insulin levels, prior to the injection of dextrose in *Mbnl3*^*ΔE2*^ mice. An increase in insulin levels was observed subsequent to dextrose injection in *Mbnl3*^+/+^ mice but not in *Mbnl3*^*ΔE2*^ mice. The lack of an increase in insulin levels subsequent to dextrose injection may be due to high, approximately a two fold elevation, in basal insulin levels in *Mbnl3*^*ΔE2*^ mice. Previous studies have strongly implicated elevated CUG-BP1 levels with aberrant glucose metabolism in DM1 patient cells^32^. Glucose intolerance is also reported in *Dmpk*^*−/−*^ animals that are maintained on a high fat diet^46^. Thus MBNL3 loss, elevated levels of CUG-BP1 and DMPK deficits can contribute to the abnormal glucose metabolism observed in DM1.

In DM1, sudden death is widely considered to result from cardiac arrhythmias and although progressive heart failure is less common, left ventricle systole dysfunction is associated with increased risk of overall mortality and sudden death^1^,^47^,^48^. Thus DM1 patients show a progressive diverse cardiac phenotype that includes both the myocardium and the conduction system^1^,^47^,^48^,^49^,^50^,^51^. Conduction system defects of sinus bradycardia, prolonged P-R and QRS intervals are commonly observed in DM1 and can occur in as many as 75% of DM1 patients. Cardiac systole dysfunction, ventricular hypertrophy and heart failure are reported in 18–50% of DM1 patients, in different studies with varying sample sizes, and can manifest with the absence of cardiac conduction defects^47^,^48^,^49^,^50^,^51^. Our results demonstrate that Mbnl3_38kD_ deficits trigger cardiac systole deficits and ventricular hypertrophy, which can be an indicator of the later onset of heart failure. EKG abnormalities are not however observed in *Mbnl3*^*ΔE2*^ mice. In contrast, our previous studies have shown that deficits of *Dmpk* and *Six5* result in conduction disorders but not echocardiographic abnormalities. Specifically, P-R prolongation, second and third degree heart block and QRS alterations are observed in *Dmpk*^+/−^, *Dmpk*^*−/−*^ and *Six5*^+/−^ mice respectively^15^,^16^,^19^. Other studies have shown that a 4–8 fold induction of CUG-BP1 in adult mouse hearts results in P-R and QRS expansion and dilated cardiomyopathy^29^. In mirror image experiments we have shown that *Mbnl1*^−/−^ loss results in arrhythmias, cardiac histopathology and sudden death and Lee *et al*. have further demonstrated that *Mbnl1*^−/−^*/Mbnl2*^+/−^ mice show both arrhythmias, left ventricular hypertrophy with myocardial fibrosis^25^,^52^. Thus the mechanistic basis of DM1 cardiac dysfunction appears to reflect composite losses of DMPK, SIX5 and the MBNL proteins in conjunction with CUG-BP1 over expression.

DM1 patients develop an unusual form of ocular cataracts that are located primarily in the posterior subcapsular and cortical regions of the lens^1^,^42^,^53^,^54^. In some patients reflective, polychromatic iridescent crystalline deposits are observed. With time dust-like opacities increase and progress to form posterior subcapsular cataracts. Other studies describe the development of cortical spokes, which can lead to complete cortical opacification. In mature DM1 cataracts, progressively larger areas are damaged and the ensuing cataracts are difficult to distinguish from the more general types of cortical cataract. Electroretinography (ERG) defects are also frequently observed with the amplitudes of both a and b waves being smaller in DM1^55^. *Mbnl3*^*ΔE2*^ mice do not show significant ERG alterations. However these animals demonstrate an increase in ocular cataracts that are subcapsular and cortical in their origin. In our study subcapsular cataracts were first detected at 7 months of age in *Mbnl3*^*ΔE2*^ mice and by 12 months of age, 100% *Mbnl3*^*ΔE2*^ lenses examined showed subcapsular or cortical cataracts, with ~60% showing advanced Grade-II and Grade-III cataracts as evaluated using LOCS II photographic grading standards. This is in contrast to *Mbnl3*^+/+^ animals, where only one of eight lenses showed grade 1 subcapsular cataracts at 12 months of age. Previous studies have demonstrated that *Mbnl1* loss results in subcapsular dust-like opacities and *Six5* deficits increase the incidence of nuclear cataracts^17^,^18^,^23^. The contribution of *Six5* deficits to the development of DM1 ocular defects is unclear, because in mice *Six5* loss results in nuclear cataracts, which are not prominent in DM1 patients. Taken together these data are consistent with Mbnl3_38kD_ and Mbnl1 deficits playing an important role in the development of the posterior subcapsular and cortical cataracts observed in DM1.

Thus, results described in this study demonstrate that Mbnl3_38kD_ deficits play a causal role in the development of a set of age-associated pathologies observed in DM1. Our results support the idea that DM1 pathology results from an aggregate of changes in *DMPK, SIX5, CUG-BP1, MBNL1, MBNL2* and *MBNL3* that occur downstream of the CTG repeat expansion. The potential synergistic interactions that result from these individual alterations and their relationship to the disease trajectory have however yet to be fully understood.

To test the molecular mechanism that underlies the onset of age-associated phenotypes in *Mbnl3*^*ΔE2*^ mice we examined splice site choice in RNAs that have been implicated in the development of DM1 pathology. Interestingly, a deficit of Mbnl3_38kD_ did not result in DM1-like splice errors in *Insr* and *Tnnt2* in muscle and heart, two splice abnormalities, which can contribute to glucose intolerance and cardiac dysfunction and hypertrophy^32^,^37^,^38^,^39^,^40^. A significant decrease in the inclusion of *Ldb3* exon 11 was however observed in *Mbnl3*^*ΔE2*^ cardiac muscle. This splice error is not reminiscent of the embryonic splice pattern, where inclusion of Ldb3 exon 11 is enhanced^33^. It is possible that the *Lbd3* splice defect observed in DM1 is an aggregate of two events, with Mbnl1 loss either overriding Mbnl3 effects or Mbnl1 loss producing larger changes than Mbnl3 loss. To more generally examine the role of Mbnl3_38kD_ depletion in the development of DM1 like splice errors we studied a range of splice events in the heart, skeletal muscle and lens of adult *Mbnl3*^+/+^ and *Mbnl3*^*ΔE2*^ mice and E18 *Mbnl3*^+/+^ embryos. No significant difference was observed for these splice events in adult *Mbnl3*^+/+^ and *Mbnl3*^*ΔE2*^ mice. These results are consistent with the observations of Poulos and colleagues who did not detect splice errors as a consequence of Mbnl3_38kD_ depletion in E18 forelimbs using RNA-seq analyses^35^. Thus, although we cannot exclude the possibility of rare DM1-like splice errors underlying the observed age-associated pathologies, our data suggest that mechanisms distinct from the adult retention of embryonic splice isoforms may make important contributions to the onset of age-associated pathologies in DM1. As noted previously, it is possible that altered levels or translation of Mbnl3_38kD_ target RNAs could underlie the phenotypes observed in *Mbnl3*^*ΔE2*^ mice^34^,^35^. Poulos and colleagues demonstrate inhibition of myogenic differentiation of muscle satellite cells leading to impaired muscle regeneration in *Mbnl3*^*ΔE2*^ mice^35^. Corresponding regenerative defects in other adult stem cells populations may contribute to the age-associated phenotypes observed in *Mbnl3*^*ΔE2*^ mice. Deciphering the molecular basis of the accelerated onset of age associated pathologies in *Mbnl3*^*ΔE2*^ mice should provide important new insights into the molecular mechanisms that contribute to DM1.

## Methods

### Ethics Statement

All experiments were performed in accordance with the institutional guidelines of both the University of Southern California (USC), and the University of California, Los Angeles (UCLA). The USC protocol was approved by the Institutional Animal Care and Use Committee at the University of Southern California, Los Angeles (Protocol number: 10347). The UCLA protocol (99-028) was approved by the UCLA Office of Animal Research Oversight.

### Ultrasound Echocardiography

Left ventricular (LV) size, mass, wall thickness, ventricular and valve function and blood flow were assessed using methods similar to those previously described^56^,^57^. Briefly, measures of chamber dimensions [end-diastolic dimension (EDD); end-systolic function (ESD); ventricular septal thickness (VST); posterior wall thickness (PWT)], heart rate, ventricular function [left ventricular % fractional shortening (LV%FS); velocity of circumferential fiber shortening (Vcf); and left ventricular ejection fraction (LVEF)] and the early (E) and atrial (A) diastolic filling (E/A ratio) were obtained from mice lightly anesthetized with isoflurane (1.0–1.5%).

### Surface ECG Recording

Electrocardiograms were obtained for at least 15 minutes from each mouse under light isoflurane anesthesia by inserting two Pt needle electrodes (Grass Technologies, USA) under the skin in the lead II configuration. The ECG data were amplified (Grass Technologies) and then digitized for analysis with HEM V4.2 software (Notocord Systems, Croissy sur Seine, France).

### Morphometry and Histology

All morphometry and histology experiments were carried out primarily as described by Jordan and colleagues^57^.

### Glucose Tolerance Testing

Glucose tolerance tests were performed according to a protocol modified from Andrikopoulos *et al*.^58^. Briefly, mice were fasted in a clean cage with water, but no food for 6 hours prior to testing. Baseline blood sugar levels were obtained from a drop of tail blood. Then a bolus injection of sterile 5% dextrose in saline was intraperitoneally injected at a dose of 1 g/kg at time zero. The blood glucose levels were repeatedly assessed for up to 3 hours following the injection and prior to feeding the mice.

### Insulin level measurements

Mice were fasted in a clean cage with water but no food for 16 hours prior to collecting blood for baseline insulin level measurements^36^. A bolus injection of sterile 20% dextrose in saline was intraperitoneally injected at a dose of 2 g/kg of body weight at time zero as previously described^36^. Blood was collected from the tail at 8, 15, 30 and 60 minutes following the dextrose injection and centrifuged to obtain clear serum. Insulin levels in the serum were measured using the mouse ultrasensitive insulin ELISA kit (ALPCO, USA) according to the manufacturer’s protocol.

### Ophthalmic examination

For ophthalmic examination of the lens, mice were first lightly anesthetized with intra-peritoneal Ketamine HCl (100 mg/kg) and the pupils were dilated with a topical application of phenylephrine hydrochloride 2.5% and tropicamide 0.5% eye drops. Slit lamp examination of the anterior segment was performed using Haag-Streit B900 slit lamp (Haag-Streit AG, Switzerland) employing direct and retroillumination techniques. A digital fundus camera system (Kowa Genesis Fundus Camera, Kowa Optimed, Inc. USA) was used for red reflex and fundus color imaging to evaluate lens clarity. Images were analyzed based on Lens Opacities Classification System-II^59^. For the ERG study, the pupils were dilated with Phenylephrine HCl (2.5%) and Tropicamide (0.5%). Anesthesia was induced with intra-peritoneal Ketamine HCl (100 mg/kg) and Xylazine HCl (10 mg/kg) with a topical corneal anesthesia of Tetracaine HCl (0.5%). Following anesthesia induction the mouse was placed in lateral recumbence on a 37 ^o^C heat therapy pad in a Faraday cage with the visual axis of the eye to be measured directed vertically. A 27 g sub-dermal platinum needle electrode was placed in the lower (ipsilateral) eyelid. Another identical (ground) electrode was placed in the (ipsilateral) ear. The tip of a carbon-fiber wick electrode was placed in contact with the cornea and all three electrodes were connected to a pre-amplifier. A fiber optic cable was centered vertically over the eye to deliver steady background and/or flashes of light (Grass PS22 photo-stimulator powering a Grass Xenon flash unit) to within several millimeters of the corneal surface. Retinal electrical responses to flash stimuli were captured on a Nicolet Electrovisual Diagnostic System for analyses.

### RNA analysis

Total RNA was prepared using Trizol (Invitrogen, USA) according to the manufacturer’s protocol. cDNA was synthesized from 5 μg of total RNA using the cDNA synthesis kit (Amersham Bioscience Inc., USA). cDNAs (150 ng) were used for splicing and qPCR studies with primers and PCR conditions described in Supplementary Table S6. For the splicing assays, PCR was performed with the indicated primers and the relative band intensities were measured by densitometry analysis using Gene Tool (Syngene Inc., USA). qPCR was performed using the CFX96 Real-time PCR Detection System (Bio-Rad) with the B-R SYBR Green Supermix (Quanta, USA). qPCR was performed to measure the relative inclusion of an alternatively spliced exon by using exon-exon boundary spanning primers that are specific to the alternatively spliced exon and to a constitutive exon. The threshold cycle (Ct) value for each alternatively spliced exon was normalized to the Ct value of the corresponding constitutive exon.

## Additional Information

**How to cite this article**: Choi, J. *et al*. Muscleblind-like 3 deficit results in a spectrum of age-associated pathologies observed in myotonic dystrophy. *Sci. Rep.* **6**, 30999; doi: 10.1038/srep30999 (2016).

## Supplementary Material

Supplementary Information
