# Supplementary material for: Muscleblind-like 3 deficit results in a spectrum of age-associated pathologies observed in myotonic dystrophy
Source: Sci Rep. 2016 Aug 3;6:30999. doi: 10.1038/srep30999 (PMC4971533; doi:10.1038/srep30999)
Supplement: Supplementary Information [file srep30999-s1.pdf]

# Muscleblind-like protein 3 deficit results in a spectrum of age-associated pathologies observed in myotonic dystrophy

*Jongkyu Choi*[1, *], *Donald M. Dixon*[1, *], *Warunee Dansithong*[1, 2, *], *Walid F. Abdallah*[3], *Kenneth P. Roos*[4], *Maria C. Jordan*[4], *Brandon Trac*[1], *Han Shin Lee*[1], *Lucio Comai*[5], *Sita Reddy*[1]

[1]Department of Biochemistry and Molecular Biology, University of Southern California, Los Angeles, CA 90033, USA; [2]Current address: Department of Neurology, Clinical Neurosciences Center, University of Utah, Salt Lake City, UT 84132, USA; [3]USC Eye Institute, Los Angeles, CA 90033, USA and Department of Ophthalmology, Faculty of Medicine, Zagazig University, Zagazig, Egypt; [4]Department of Physiology, David Geffen School of Medicine at UCLA, Los Angeles, CA 90095-1751, USA. [5]Department of Microbiology and Immunology, University of Southern California, Los Angeles, CA 90033, USA.

* These authors contributed equally to the study.

Address correspondence to Sita Reddy at Institute for Genetic Medicine, CSC-240, Keck School of Medicine, University of Southern California, 2250 Alcazar Street, Los Angeles, CA 90033, USA. Email: sitaredd@usc.edu

Running Title: Age-associated pathologies in *Mbnl3* deficient mice

## SUPPLEMENTARY FIGURE AND TABLE LEGENDS

**Supplementary Fig. S1. RT-PCR analysis of *Mbnl3* expression in adult mouse tissues.**

RNA samples from 129Sv *Mbnl3*$^{+/+}$ and 129Sv *Mbnl3*$^{\Delta E2}$ E18 placenta and the indicated adult tissues/organs were subjected to RT-PCR (40 cycles) using primers located in Mbnl3 exon 2.

**Supplementary Fig. S2. Splicing analysis in *Mbnl3*$^{\Delta E2}$ soleus muscle and heart.**

**a.** *Insr* exon 10a inclusion was not significantly different in *Mbnl3*$^{+/+}$ and *Mbnl3*$^{\Delta E2}$ muscles at 7 months of age. Alternative splicing was analyzed for *Insr* by RT-PCR in male *Mbnl3*$^{+/+}$ and male *Mbnl3*$^{\Delta E2}$ soleus muscles at 7 months of age. Band intensities were quantitated by densitometry. **b.** Splicing analyses for *Ldb3, Tnnt2, Insr* and *Mbnl1* performed by RT-PCR in male *Mbnl3*$^{+/+}$ and male *Mbnl3*$^{\Delta E2}$ hearts at 7 months of age. *Tnnt2* exon 4 + 5, *Insr* exon 10a and *Mbnl1* exon 7 inclusion was not significantly different in *Mbnl3*$^{+/+}$ and *Mbnl3*$^{\Delta E2}$ hearts at 7 months of age. *Ldb3* exon 11 inclusion in *Mbnl3*$^{\Delta E}$ hearts was significantly decreased when compared to *Mbnl3*$^{+/+}$ hearts at 7 months of age. **c.** *Mbnl1* exon 7 and *Insr* exon 10a inclusion was not significantly different in male *Mbnl3*$^{+/+}$ and *Mbnl3*$^{\Delta E}$ hearts at 11 months of age. Alternative splicing was analyzed for *Mbnl1* and *Insr* by RT-PCR in male *Mbnl3*$^{+/+}$ and male *Mbnl3*$^{\Delta E2}$ hearts at 11 months of age. Band intensities were quantitated by densitometry. The exon numbers are indicated and the alternatively spliced exons are shown as a gray box. Exon numbers are annotated based on Refseq from UCSC genome browser (NCBI37/mm9). The expected band sizes and p-values are shown. Data are standard error of mean.

**Supplementary Fig. S3. Splicing analysis in *Mbnl3*$^{\Delta E2}$ hearts at 11 months of age.**

Splicing analyses for *Pdlim3/Alp, Trim55/Murf2, Mapt/Tau, Pdlim5, Sorbs1, Sorbs2, Fhod1, Spag9, Mbnl2, Myom1, Clta, Stx2, Csda, Sirt2, Atp2a1, Atp11a and Gapdh* were performed by RT-PCR in male *Mbnl3*$^{+/+}$ and male *Mbnl3*$^{\Delta E2}$ hearts (n=3/genotype) at 11 months of age and E18 *Mbnl3*$^{+/+}$ hearts. Band intensities were quantitated by densitometry. The exon numbers are indicated and the alternatively spliced exons are shown as a green box. Exon numbers are annotated based on Refseq from UCSC genome browser (NCBI37/mm9). The expected band sizes and p-values are shown. Data are standard error of mean.

**Supplementary Fig. S4. Splicing analysis in *Mbnl3*$^{\Delta E2}$ soleus muscles at 11 months of age.**

Splicing analyses for *Pdlim3/Alp, Trim55/Murf2, Mapt/Tau, Pdlim5, Sorbs1, Sorbs2, Fhod1, Spag9, Mbnl2, Myom1, Clta, Stx2, Csda, Sirt2, Atp2a1, Atp11a and Gapdh* were performed by RT-PCR in male *Mbnl3*$^{+/+}$ and male *Mbnl3*$^{\Delta E2}$ soleus muscles (n=3/genotype) at 11 months of age and E18 *Mbnl3*$^{+/+}$ forelimbs. Band intensities were quantitated by densitometry. The exon numbers are indicated and the alternatively spliced exons are shown as a green box. Exon numbers are annotated based on Refseq from UCSC genome browser (NCBI37/mm9). The expected band sizes and p-values are shown. Data are standard error of mean.

**Supplementary Fig. S5. Splicing analysis in *Mbnl3*$^{\Delta E2}$ whole lens at 8 months of age.**

Splicing analyses for *Mapt/Tau, Pdlim5, Sorbs1, Sorbs2, Spag9, Mbnl2, Stx2, Csda, Sirt2, Atp11a and Gapdh* were performed by RT-PCR in the whole lens from male *Mbnl3*$^{+/+}$ and male *Mbnl3*$^{\Delta E2}$ mice (n=3/genotype) at 8 months of age and E18 *Mbnl3*$^{+/+}$ mice. Band intensities were quantitated by densitometry. The exon numbers are indicated and the alternatively spliced exons are shown as a green box. Exon numbers are annotated based on Refseq from UCSC genome

browser (NCBI37/mm9). The expected band sizes and p-values are shown. Data are standard error of mean.

**Supplementary Table S1. a & b.** Blood glucose levels (mg/dL) subsequent to dextrose injection at time 0 for male *Mbnl3*$^{+/+}$ and male *Mbnl3*$^{\Delta E2}$ mice at 4 and 7-9 months of age are shown. p-values were calculated using the Student's t-test with significance set at $p \leq 0.05$. Values where $p \leq 0.05$ are shown in red. Data are standard error of mean.

**Supplementary Table S2.** Blood insulin levels (ng/ml) subsequent to dextrose injection at time 0 for male *Mbnl3*$^{+/+}$ and male *Mbnl3*$^{\Delta E2}$ mice at 8-10 months of age are shown. p-values were calculated using the Student's t-test with significance set at $p \leq 0.05$. Values where $p \leq 0.05$ are shown in red. Data are standard error of mean.

**Supplementary Table S3. a & b.** Ultrasound echocardiographic values recorded from male *Mbnl3*$^{+/+}$ and male *Mbnl3*$^{\Delta E2}$ mice at 4 months and 11 months of age are shown. Measurements of the left ventricle taken in this study include ventricular septal thickness (VST), end-diastolic dimension (EDD), posterior wall thickness (PWT), end-systolic dimension (ESD), and left ventricle mass (Lvmass). Heart function assessments included aorta ejection time (Ao-ET), left ventricle percent fractional shortening (Lv%FS), velocity of circumferential fiber shortening (Vcf), left ventricle ejection fraction (LvEF) and the E/A ratio. Data are mean and standard deviation. p-values were calculated using the Student's t-test with significance set at $p \leq 0.05$. Values where $p \leq 0.05$ are shown in red.

**Supplementary Table S4. a & b**. EKG values for male *Mbnl3*$^{+/+}$ and male *Mbnl3*$^{\Delta E2}$ mice at 4 and 11 months of age are shown. Data are mean and standard deviation. p-values were calculated using the Student's t-test with significance set at $p \leq 0.05$.

**Supplementary Table S5.** Electroretinography values for male *Mbnl3*$^{+/+}$ and male *Mbnl3*$^{\Delta E2}$ mice at 13 months of age are shown. Data are mean and standard deviation. p-values were calculated using the Student's t-test with significance set at $p \leq 0.05$.

**Supplementary Table S6.** PCR conditions and primers.

Figure S1

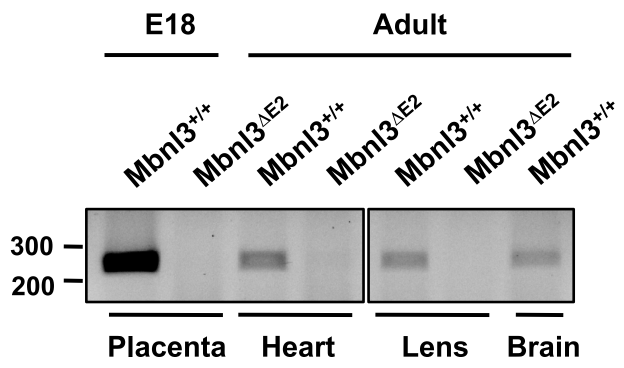

E18
Adult
Mbnl3+/+
Mbnl3ΔE2
Mbnl3+/+
Mbnl3ΔE2
Mbnl3+/+
Mbnl3ΔE2
Mbnl3+/+
300
200
Placenta
Heart
Lens
Brain

Figure S2

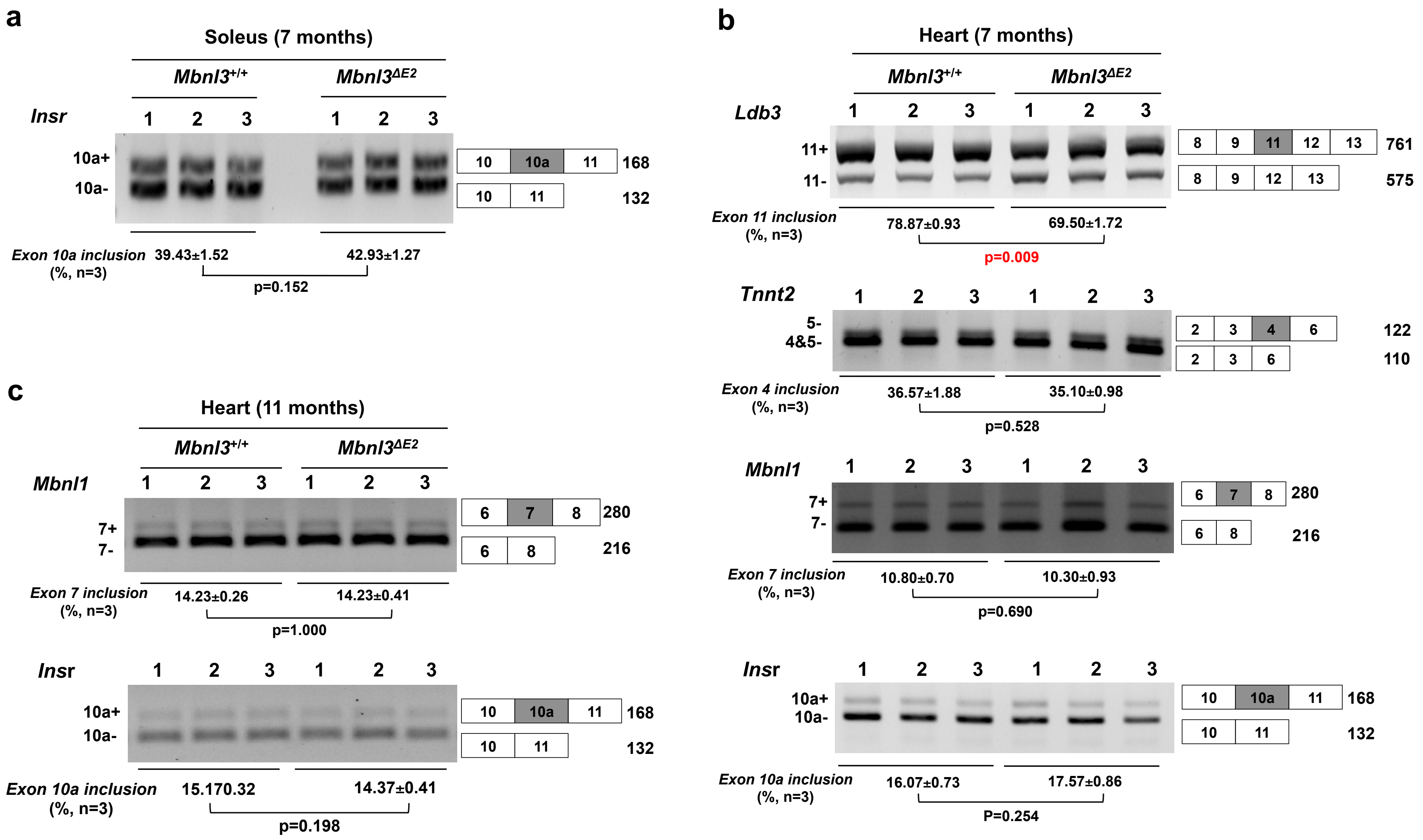

a
Soleus (7 months)
Mbnl3+/+
Mbnl3ΔE2
Insr
1 2 3 1 2 3
10a+
10a-
10 10a 11 168
10 11 132
Exon 10a inclusion (%, n=3)
39.43±1.52
42.93±1.27
p=0.152
b
Heart (7 months)
Mbnl3+/+
Mbnl3ΔE2
Ldb3
1 2 3 1 2 3
11+
11-
8 9 11 12 13 761
8 9 12 13 575
Exon 11 inclusion (%, n=3)
78.87±0.93
69.50±1.72
p=0.009
Tnnt2
1 2 3 1 2 3
5-
4&5-
2 3 4 6 122
2 3 6 110
Exon 4 inclusion (%, n=3)
36.57±1.88
35.10±0.98
p=0.528
Mbnl1
1 2 3 1 2 3
7+
7-
6 7 8 280
6 8 216
Exon 7 inclusion (%, n=3)
10.80±0.70
10.30±0.93
p=0.690
Insr
1 2 3 1 2 3
10a+
10a-
10 10a 11 168
10 11 132
Exon 10a inclusion (%, n=3)
16.07±0.73
17.57±0.86
P=0.254
c
Heart (11 months)
Mbnl3+/+
Mbnl3ΔE2
Mbnl1
1 2 3 1 2 3
7+
7-
6 7 8 280
6 8 216
Exon 7 inclusion (%, n=3)
14.23±0.26
14.23±0.41
p=1.000
Insr
1 2 3 1 2 3
10a+
10a-
10 10a 11 168
10 11 132
Exon 10a inclusion (%, n=3)
15.170.32
14.37±0.41
p=0.198

Figure S3

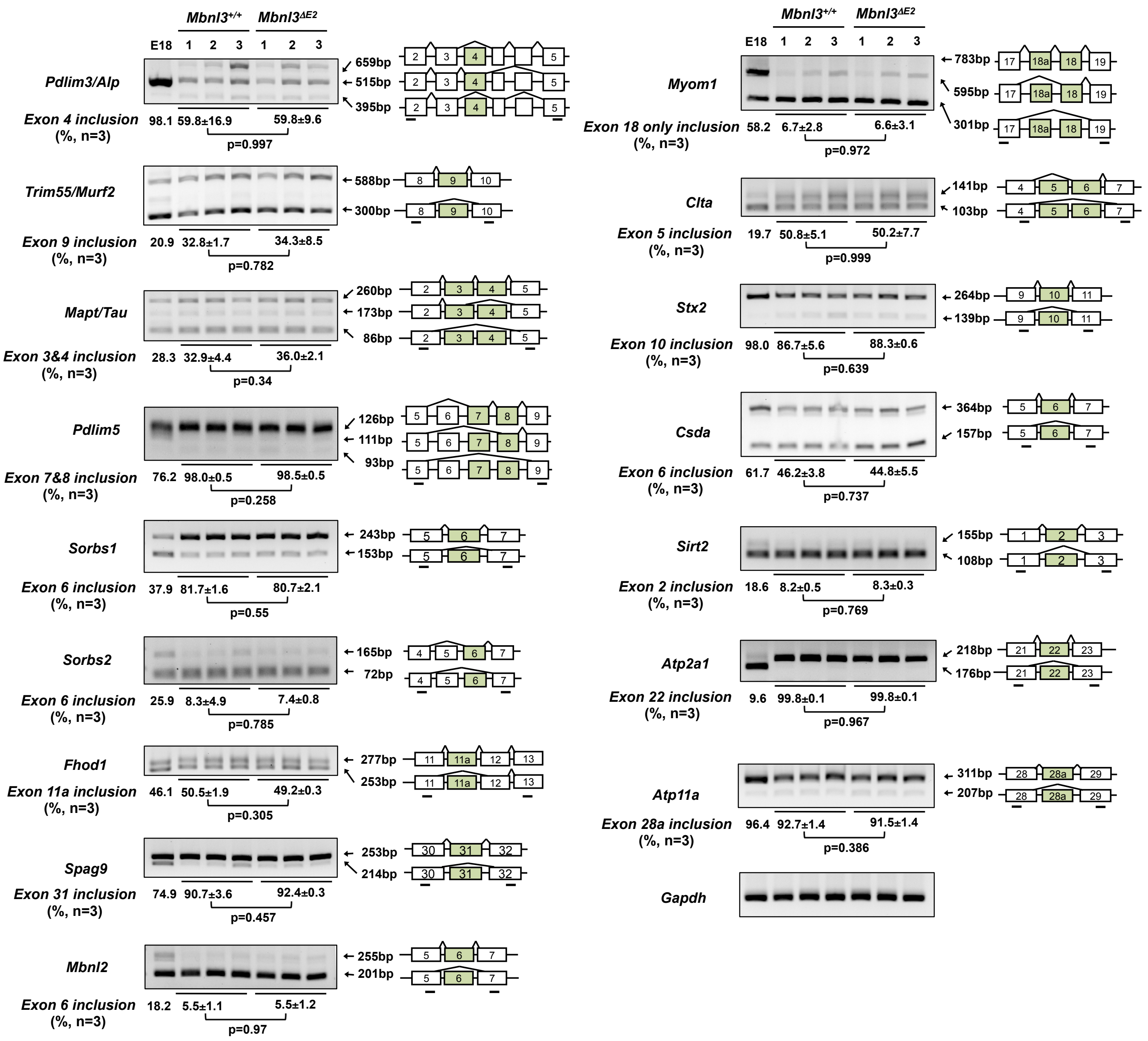

Mbnl3+/+
Mbnl3ΔE2
E18 1 2 3 1 2 3
Pdlim3/Alp
659bp
515bp
395bp
Exon 4 inclusion (%, n=3)
98.1 59.8±16.9 59.8±9.6
p=0.997
Trim55/Murf2
588bp
300bp
Exon 9 inclusion (%, n=3)
20.9 32.8±1.7 34.3±8.5
p=0.782
Mapt/Tau
260bp
173bp
86bp
Exon 3&4 inclusion (%, n=3)
28.3 32.9±4.4 36.0±2.1
p=0.34
Pdlim5
126bp
111bp
93bp
Exon 7&8 inclusion (%, n=3)
76.2 98.0±0.5 98.5±0.5
p=0.258
Sorbs1
243bp
153bp
Exon 6 inclusion (%, n=3)
37.9 81.7±1.6 80.7±2.1
p=0.55
Sorbs2
165bp
72bp
Exon 6 inclusion (%, n=3)
25.9 8.3±4.9 7.4±0.8
p=0.785
Fhod1
277bp
253bp
Exon 11a inclusion (%, n=3)
46.1 50.5±1.9 49.2±0.3
p=0.305
Spag9
253bp
214bp
Exon 31 inclusion (%, n=3)
74.9 90.7±3.6 92.4±0.3
p=0.457
Mbnl2
255bp
201bp
Exon 6 inclusion (%, n=3)
18.2 5.5±1.1 5.5±1.2
p=0.97
Myom1
783bp
595bp
301bp
Exon 18 only inclusion (%, n=3)
58.2 6.7±2.8 6.6±3.1
p=0.972
Clta
141bp
103bp
Exon 5 inclusion (%, n=3)
19.7 50.8±5.1 50.2±7.7
p=0.999
Stx2
264bp
139bp
Exon 10 inclusion (%, n=3)
98.0 86.7±5.6 88.3±0.6
p=0.639
Csda
364bp
157bp
Exon 6 inclusion (%, n=3)
61.7 46.2±3.8 44.8±5.5
p=0.737
Sirt2
155bp
108bp
Exon 2 inclusion (%, n=3)
18.6 8.2±0.5 8.3±0.3
p=0.769
Atp2a1
218bp
176bp
Exon 22 inclusion (%, n=3)
9.6 99.8±0.1 99.8±0.1
p=0.967
Atp11a
311bp
207bp
Exon 28a inclusion (%, n=3)
96.4 92.7±1.4 91.5±1.4
p=0.386
Gapdh

Figure S4

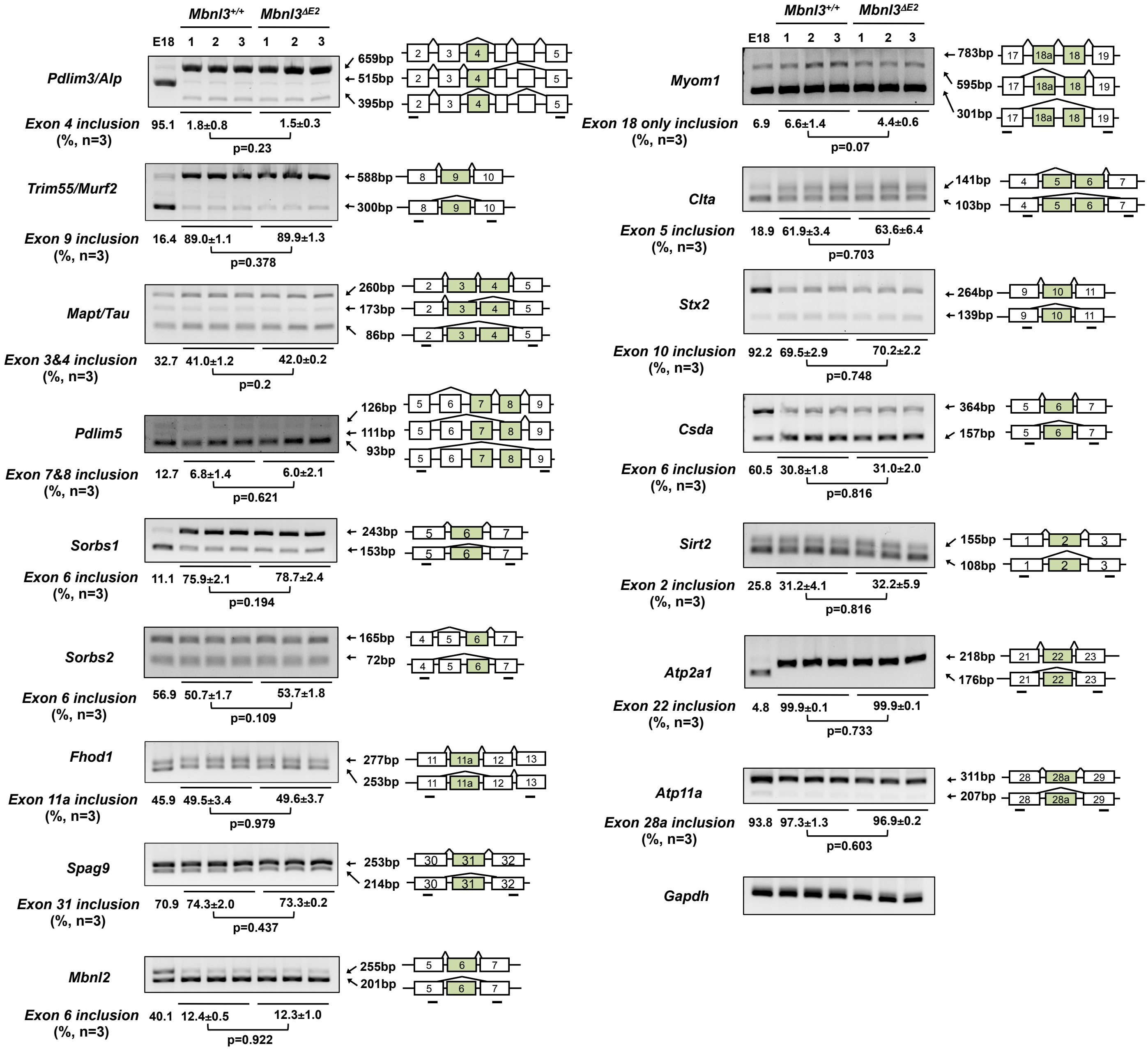
Mbnl3+/+
Mbnl3ΔE2
E18 1 2 3 1 2 3
Pdlim3/Alp
659bp
515bp
395bp
Exon 4 inclusion (%, n=3)
95.1 1.8±0.8 1.5±0.3
p=0.23
Trim55/Murf2
588bp
300bp
Exon 9 inclusion (%, n=3)
16.4 89.0±1.1 89.9±1.3
p=0.378
Mapt/Tau
260bp
173bp
86bp
Exon 3&4 inclusion (%, n=3)
32.7 41.0±1.2 42.0±0.2
p=0.2
Pdlim5
126bp
111bp
93bp
Exon 7&8 inclusion (%, n=3)
12.7 6.8±1.4 6.0±2.1
p=0.621
Sorbs1
243bp
153bp
Exon 6 inclusion (%, n=3)
11.1 75.9±2.1 78.7±2.4
p=0.194
Sorbs2
165bp
72bp
Exon 6 inclusion (%, n=3)
56.9 50.7±1.7 53.7±1.8
p=0.109
Fhod1
277bp
253bp
Exon 11a inclusion (%, n=3)
45.9 49.5±3.4 49.6±3.7
p=0.979
Spag9
253bp
214bp
Exon 31 inclusion (%, n=3)
70.9 74.3±2.0 73.3±0.2
p=0.437
Mbnl2
255bp
201bp
Exon 6 inclusion (%, n=3)
40.1 12.4±0.5 12.3±1.0
p=0.922
Myom1
783bp
595bp
301bp
Exon 18 only inclusion (%, n=3)
6.9 6.6±1.4 4.4±0.6
p=0.07
Clta
141bp
103bp
Exon 5 inclusion (%, n=3)
18.9 61.9±3.4 63.6±6.4
p=0.703
Stx2
264bp
139bp
Exon 10 inclusion (%, n=3)
92.2 69.5±2.9 70.2±2.2
p=0.748
Csda
364bp
157bp
Exon 6 inclusion (%, n=3)
60.5 30.8±1.8 31.0±2.0
p=0.816
Sirt2
155bp
108bp
Exon 2 inclusion (%, n=3)
25.8 31.2±4.1 32.2±5.9
p=0.816
Atp2a1
218bp
176bp
Exon 22 inclusion (%, n=3)
4.8 99.9±0.1 99.9±0.1
p=0.733
Atp11a
311bp
207bp
Exon 28a inclusion (%, n=3)
93.8 97.3±1.3 96.9±0.2
p=0.603
Gapdh

Figure S5

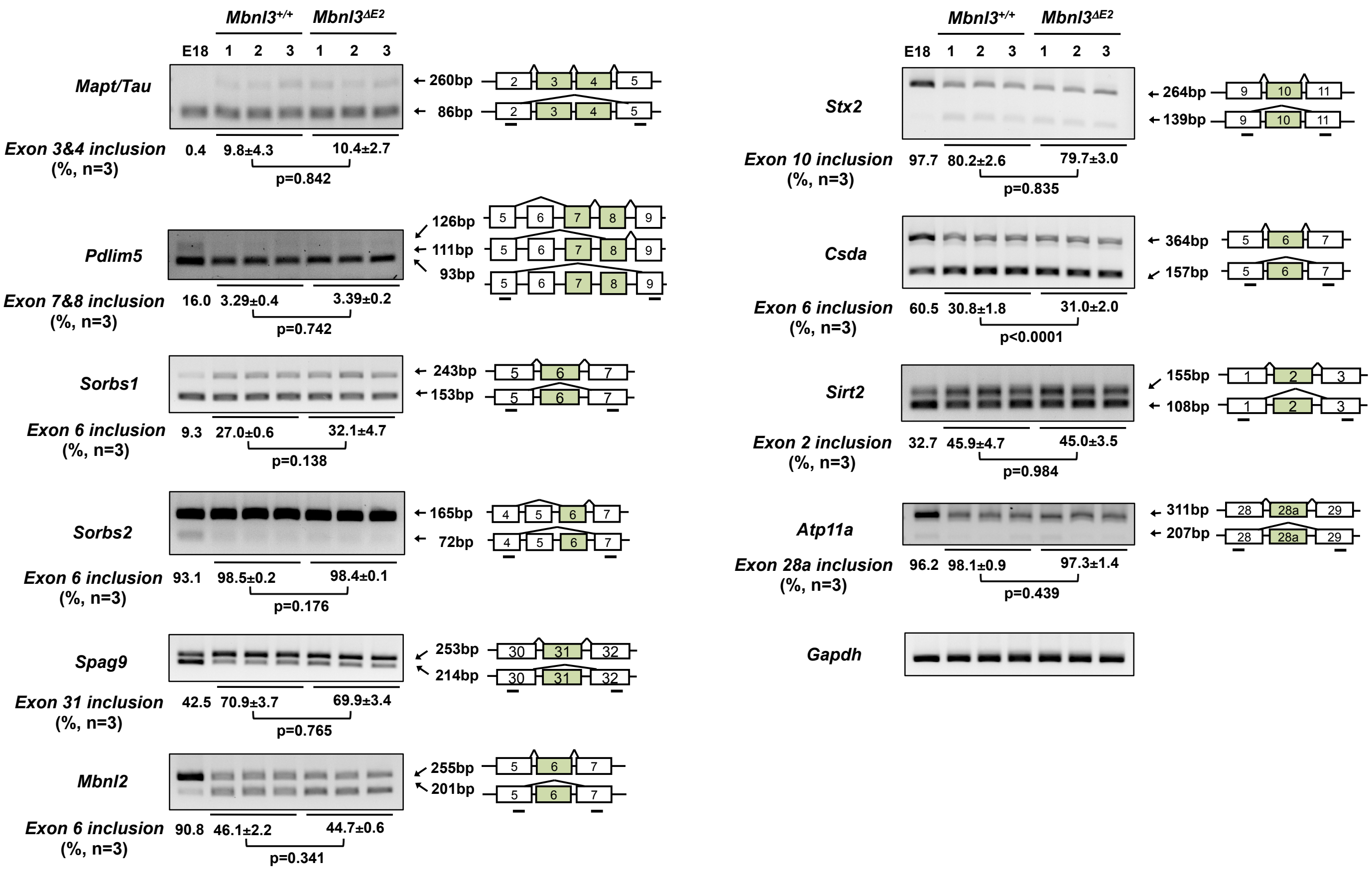
Mbnl3+/+
Mbnl3ΔE2
E18 1 2 3 1 2 3
Mapt/Tau
260bp
86bp
Exon 3&4 inclusion (%, n=3)
0.4 9.8±4.3 10.4±2.7
p=0.842
Pdlim5
126bp
111bp
93bp
Exon 7&8 inclusion (%, n=3)
16.0 3.29±0.4 3.39±0.2
p=0.742
Sorbs1
243bp
153bp
Exon 6 inclusion (%, n=3)
9.3 27.0±0.6 32.1±4.7
p=0.138
Sorbs2
165bp
72bp
Exon 6 inclusion (%, n=3)
93.1 98.5±0.2 98.4±0.1
p=0.176
Spag9
253bp
214bp
Exon 31 inclusion (%, n=3)
42.5 70.9±3.7 69.9±3.4
p=0.765
Mbnl2
255bp
201bp
Exon 6 inclusion (%, n=3)
90.8 46.1±2.2 44.7±0.6
p=0.341
Stx2
264bp
139bp
Exon 10 inclusion (%, n=3)
97.7 80.2±2.6 79.7±3.0
p=0.835
Csda
364bp
157bp
Exon 6 inclusion (%, n=3)
60.5 30.8±1.8 31.0±2.0
p<0.0001
Sirt2
155bp
108bp
Exon 2 inclusion (%, n=3)
32.7 45.9±4.7 45.0±3.5
p=0.984
Atp11a
311bp
207bp
Exon 28a inclusion (%, n=3)
96.2 98.1±0.9 97.3±1.4
p=0.439
Gapdh

## Table S1

**a**

| Genotype | Gender | Age (months) | n | 0 min | 10 mins | 20 mins | 30 mins | 60 mins | 90 mins | 120 mins | 180 mins |
|---|---|---|---|---|---|---|---|---|---|---|---|
| ***Mbnl3***$^{+/+}$ | Male | 4 | 4 | 93.13±22.99 | 164.00±26.75 | 179.88±37.84 | 168.13±37.60 | 123.50±30.18 | 123.38±19.60 | 108.00±21.04 | 88.50±16.69 |
| ***Mbnl3***$^{\Delta E2}$ | Male | 4 | 3 | 101.33±15.67 | 179.67±17.75 | 143.67±45.17 | 126.33±35.11 | 128.50±17.21 | 117.83±23.06 | 123.67±30.15 | 113.20±25.25 |
| **p-value** | | | | 0.468 | 0.239 | 0.128 | 0.056 | 0.732 | 0.636 | 0.273 | 0.083 |

**b**

| Genotype | Gender | Age (months) | n | 0 min | 10 mins | 20 mins | 30 mins | 60 mins | 90 mins | 120 mins | 180 mins |
|---|---|---|---|---|---|---|---|---|---|---|---|
| ***Mbnl3***$^{+/+}$ | Male | 7-9 | 5 | 74.72±4.87 | 134.64±11.21 | 152.80±4.97 | 99.20±7.27 | 92.09±3.08 | 77.70±4.72 | 76.90±6.74 | 62.20±5.62 |
| ***Mbnl3***$^{\Delta E2}$ | Male | 7-9 | 7 | 111.88±3.33 | 163.13±8.53 | 191.43±14.88 | 178.64±14.88 | 164.72±12.50 | 142.57±7.65 | 130.57±7.46 | 108.40±3.90 |
| **p-value** | | | | *<0.001 | *0.021 | *0.045 | *<0.001 | *<0.001 | *<0.001 | *<0.001 | *<0.001 |

## Table S2

| Genotype | Gender | Age (months) | n | 0 min | 8 mins | 15 mins | 30 mins | 60 mins | 90 mins |
|---|---|---|---|---|---|---|---|---|---|
| ***Mbnl3***$^{+/+}$ | Male | 8-10 | 4 | 0.74±40.10 | 1.29±0.12 | 1.02±0.09 | 1.08±0.13 | 0.86±0.09 | 0.85±0.06 |
| ***Mbnl3***$^{\Delta E2}$ | Male | 8-10 | 5 | 1.62±0.21 | 1.78±0.21 | 1.58±0.19 | 1.93±0.20 | 1.76±0.23 | 1.81±0.25 |
| **p-value** | | | | *0.005 | 0.275 | *0.013 | *0.048 | *0.014 | *0.008 |

**Table S3**

**a**

| Genotype | Gender | Age (weeks) | n | Heart Rate | VST (mm) | EDD (mm) | PWT (mm) | ESD (mm) | Ao-ET (ms) | Lv%FS | Vcf | LvEF | Lvmass | E/A |
|---|---|---|---|---|---|---|---|---|---|---|---|---|---|---|
| ***Mbnl3***$^{+/+}$ | Male | 17 | 4 | 476±64 | 0.55±0.01 | 4.25±0.10 | 0.54±0.05 | 2.68±0.25 | 54.3±2.1 | 37.0±5.7 | 6.80±0.86 | 73.8±7.7 | 78.1±3.5 | 1.81±0.15 |
| ***Mbnl3***$^{\Delta E2}$ | Male | 17 | 7 | 475±48 | 0.56±0.04 | 4.20±0.23 | 0.50±0.05 | 3.04±0.21 | 52.1±3.4 | 27.5±4.6 | 5.29±0.87 | 59.9±7.8 | 76.0±11.2 | 2.10±0.65 |
| **p-value** | | | | 0.97 | 0.64 | 0.72 | 0.3 | ***0.03** | 0.26 | ***0.01** | ***0.02** | ***0.02** | 0.72 | 0.48 |

**b**

| Genotype | Gender | Age (weeks) | n | Heart Rate | VST (mm) | EDD (mm) | PWT (mm) | ESD (mm) | Ao-ET (ms) | Lv%FS | Vcf | LvEF | Lvmass | E/A |
|---|---|---|---|---|---|---|---|---|---|---|---|---|---|---|
| ***Mbnl3***$^{+/+}$ | Male | 46 | 4 | 468±50 | 0.46±0.02 | 4.00±0.20 | 0.46±0.01 | 2.85±0.44 | 48.9±4.6 | 29.0±7.8 | 5.98±1.69 | 63.9±9.9 | 58.7±6.9 | 1.50±0.16 |
| ***Mbnl3***$^{\Delta E2}$ | Male | 46 | 6 | 525±38 | 0.53±0.04 | 4.48±0.35 | 0.56±0.05 | 3.37±0.42 | 47.1±8.1 | 24.1±6.0 | 5.19±1.45 | 55.6±9.9 | 88.5±11.2 | 1.79±0.35 |
| **p-value** | | | | 0.07 | ***0.02** | ***0.04** | ***0.003** | 0.09 | 0.69 | 0.37 | 0.57 | 0.22 | ***0.001** | 0.16 |

# Table S4

**a**

| Genotype | Gender | Age (weeks) | n | PR (ms) | QRS (ms) | QT (ms) |
|---|---|---|---|---|---|---|
| ***Mbnl3+/+*** | Male | 17 | 4 | 42.9±2.2 | 8.9±0.8 | 45.8±1.8 |
| ***Mbnl3$^{\Delta E2}$*** | Male | 17 | 4 | 45.2±2.3 | 8.3±0.2 | 51.0±7.1 |
| **p-value** | | | | 0.198 | 0.199 | 0.228 |

**b**

| Genotype | Gender | Age (weeks) | n | PR (ms) | QRS (ms) | QT (ms) |
|---|---|---|---|---|---|---|
| ***Mbnl3+/+*** | Male | 46 | 4 | 48.9±3.4 | 10.0±3.4 | 49.9±0.9 |
| ***Mbnl3$^{\Delta E2}$*** | Male | 46 | 4 | 44.2±1.0 | 9.7±2.2 | 55.8±4.1 |
| **p-value** | | | | 0.085 | 0.908 | 0.068 |

# Table S5

| Genotype | Gender | Age (months) | n | Scotopic Max (µV) | | Mesopic (µV) | | Photopic (µV) | | | | | | | | |
|---|---|---|---|---|---|---|---|---|---|---|---|---|---|---|---|---|
| | | | | | | | | 1 min | | 4 min | | 7 min | | 10 min | | 10 Hz |
| | | | | a-wave | b-wave | a-wave | b-wave | a-wave | b-wave | a-wave | b-wave | a-wave | b-wave | a-wave | b-wave | Flicker |
| ***Mbnl3*** $^{\Delta E2}$ | Male | 13 | 4 | 51.8±13.7 | 640.5±175.1 | 419.0±50.8 | 1065.0±357.9 | 21.3±4.3 | 288.3±31.7 | 19.5±13.9 | 310.0±77.4 | 23.8±5.4 | 336.8±83.0 | 26.8±8.6 | 350.3±85.0 | 144.5±70.6 |
| ***Mbnl3*** $^{+/+}$ | Male | 13 | 4 | 36.0±16.1 | 734.0±204.8 | 370.5±69.1 | 1234.3±249.2 | 18.3±3.2 | 262.3±68.0 | 21.5±1.7 | 277.0±69.6 | 21.8±3.1 | 287.8±54.8 | 17.8±2.9 | 292.8±65.4 | 109.5±26.9 |
| **p-value** | | | | 0.187 | 0.514 | 0.301 | 0.467 | 0.371 | 0.524 | 0.785 | 0.549 | 0.543 | 0.363 | 0.095 | 0.325 | 0.39 |

**Table S6**

## *PCR primers and conditions:*

| RT-PCR primers | PCR Conditions: |
|---|---|
| **Muscleblind-like protein 3 (*Mbnl3*)** | |
| Forward: 5'-TCCTTGAACCATCTGCAGTCA-3' | 5 MIN AT 95C, [30 SEC AT 95C, 30 SEC AT 60C, |
| Reverse: 5'-GTGAATCAAAACAGGCCACCA-3' | 45 SEC AT 72C X35], 10 MIN AT 72C |
| **Insulin receptor (*Insr*)** | |
| Forward: 5'-GAGGATTACCTGCACAACG-3' | 5 MIN AT 95C, [30 SEC AT 95C, 30 SEC AT 55C, |
| Reverse: 5'-CACAATGGTAGAGGAGACG-3' | 45 SEC AT 72C X35], 10 MIN AT 72C |
| **Cardiac troponin T (*Tnnt2*)** | |
| Forward: 5'-GCCGAGGAGGTGGTGGAGGAGTA-3' | 5 MIN AT 95C, [30 SEC AT 95C, 30 SEC AT 65C, |
| Reverse: 5'-GTCTCAGCCTCACCCTCAGGCTCA-3' | 45 SEC AT 72C X35], 10 MIN AT 72C |
| **LIM domain binding protein 3 (*Ldb3*)** | |
| Forward: 5'-GGAAGATGAGGCTGATGAGTGG-3' | 5 MIN AT 95C, [30 SEC AT 95C, 30 SEC AT 60C, |
| Reverse: 5'-TGCTGACAGTGGTAGTGCTCTTTC-3' | 45 SEC AT 72C X35], 10 MIN AT 72C |
| **Muscleblind-like protein 1 (*Mbnl1*)** | |
| Forward: 5'-GCTGCCCAATACCAGGTCAAC-3' | 5 MIN AT 95C, [30 SEC AT 95C, 30 SEC AT 60C, |
| Reverse: 5'-TGGTGGGAGAAATGCTGTATGC-3' | 45 SEC AT 72C X35], 10 MIN AT 72C |
| **PDZ and LIM domain 5 (*Pdlim3/Alp*)** | |
| Forward: 5'- AGC TGC CAA CCT GTG TCC TG -3' | 5 MIN AT 95C, [30 SEC AT 95C, 30 SEC AT 60C, |
| Reverse: 5'- GAT CCT GCA GCA CCC TGA AG -3 | 45 SEC AT 72C X35], 10 MIN AT 72C |
| **ATPase, Ca++ transporting, cardiac muscle, fast twitch 1 *(Atp2a1)*** | |
| Forward: 5'- GCT CAT GGT CCT CAA GAT CTC AC -3' | 5 MIN AT 95C, [30 SEC AT 95C, 30 SEC AT 60C, |
| Reverse: 5'- GGG TCA GTG CCT CAG CTT TG -3 | 45 SEC AT 72C X35], 10 MIN AT 72C |
| **Clathrin, light chain A (*Clta*)** | |
| Forward: 5'- GGC GAT AAA GGA GCT GGA AGA -3' | 5 MIN AT 95C, [30 SEC AT 95C, 30 SEC AT 60C, |
| Reverse: 5'- AAT GTC GTT TAC AAA GGC TTC -3' | 45 SEC AT 72C X35], 10 MIN AT 72C |
| **Cold shock domain protein A/Y-box binding portein 3 (*Csda/Ybx3*)** | |
| Forward: 5'- GGT GCT GAA GCA GCA AAC GT -3' | 5 MIN AT 95C, [30 SEC AT 95C, 30 SEC AT 60C, |
| Reverse: 5'- GGA CTC CAT CCT TCA TCT CCC C -3' | 45 SEC AT 72C X35], 10 MIN AT 72C |

***Continued***

| **RT-PCR primers** | **PCR Conditions:** |
| --- | --- |
| **Muscleblind-like protein 2 (*Mbnl2*)** | |
| Forward: 5’- ACC GTA ACC GTT TGT ATG GAT TAC -3’ | 5 MIN AT 95C, [30 SEC AT 95C, 30 SEC AT 60C, |
| Reverse: 5’- CTT TGG TAA GGG ATG AAG AGC AC -3’ | 45 SEC AT 72C X35], 10 MIN AT 72C |
| **Myomesin 1 *(Myom1*)** | |
| Forward: 5’- CAT GGA CTG ACG ACT GCT CAG AGC -3’ | 5 MIN AT 95C, [30 SEC AT 95C, 30 SEC AT 55C, |
| Reverse: 5’- CTG CAT CGC TGA CGG CCT TGA TG -3’ | 45 SEC AT 72C X35], 10 MIN AT 72C |
| **Sorbin and SH3 domain-containing protein 2 (*Sorbs2*)** | |
| Forward: 5’- CTG AAG AAG TTA ATA ACA AAC CCT TCA AGG TG -3’ | 5 MIN AT 95C, [30 SEC AT 95C, 30 SEC AT 65C, |
| Reverse: 5’- CTG AAT CTG GAG ACT GAG ACT CAC G -3’ | 45 SEC AT 72C X35], 10 MIN AT 72C |
| **Syntaxin 2 (S*tx2*)** | |
| Forward: 5’- GCC AAG GAA GAG ACG AAG AAA GC -3’ | 5 MIN AT 95C, [30 SEC AT 95C, 30 SEC AT 60C, |
| Reverse: 5’-GGT ACG GTT GCT ATG ACA ATG CTG -3’ | 45 SEC AT 72C X35], 10 MIN AT 72C |
| **Tripartite motif containing 55 (*Trim55/Murf-2*)** | |
| Forward: 5’- GGA GAG GAT GCA GTA GAA GTA -3’ | 5 MIN AT 95C, [30 SEC AT 95C, 30 SEC AT 60C, |
| Reverse: 5’- CAA CCA GGA GAA GGA GAA GAC -3’ | 45 SEC AT 72C X35], 10 MIN AT 72C |
| ***ATPase , class VI, type 11A (Atp11a)*** | |
| Forward: 5’- CCG TTC CTC AGT TAC CAG AGG AT -3’ | 5 MIN AT 95C, [30 SEC AT 95C, 30 SEC AT 60C, |
| Reverse: 5’- CAG AGG GGT GAA TTC TGA AAT GCT G -3’ | 45 SEC AT 72C X35], 10 MIN AT 72C |
| **Formin homolog 2 domain containing 1 (*Fhod1)*** | |
| Forward: 5’- CGC CTA CAA ATC CAG CCT TC -3’ | 5 MIN AT 95C, [30 SEC AT 95C, 30 SEC AT 60C, |
| Reverse: 5’- TCC CTT GTG CCT GAT GAT CC -3’ | 45 SEC AT 72C X35], 10 MIN AT 72C |
| **PDZ and LIM domain 5 (*Pdlim5*)** | |
| Forward: 5’- CAG GGT GAC ATT AAG CAG CAA AAT GG -3’ | 5 MIN AT 95C, [30 SEC AT 95C, 30 SEC AT 60C, |
| Reverse: 5’- GGC ATC ACT GTG AGT GGG TAT GTG -3’ | 45 SEC AT 72C X35], 10 MIN AT 72C |
| **Sirtuin 2 (*Sirt2*)** | |
| Forward: 5’- CAG AGC AGT CGG TGA CAG TCC -3’ | 5 MIN AT 95C, [30 SEC AT 95C, 30 SEC AT 60C, |
| Reverse: 5’- TCT CTG CCT CTC CAC CAG TG -3’ | 45 SEC AT 72C X35], 10 MIN AT 72C |

***Continued***

| **RT-PCR primers** | **PCR Conditions:** |
|---|---|
| **Sorbin and SH3 domain containing 1 (*Sorbs1*)** | |
| Forward: 5'- CTG GCA ACC ATG GAC CAG AC -3'<br>Reverse: 5'- CTG ACT TGC TTT CAT GCT TCG -3 | 5 MIN AT 95C, [30 SEC AT 95C, 30 SEC AT 60C,<br>45 SEC AT 72C X35], 10 MIN AT 72C |
| **C-jun-amino-terminal kinase-interacting protein (*Spag9*)** | |
| Forward: 5'- GGA CTG GAA ATG GTGTCATTA TCT CCA T -3'<br>Reverse: 5'-ACT GCC ACA AAG AAT TTC ACA G -3' | 5 MIN AT 95C, [30 SEC AT 95C, 30 SEC AT 55C,<br>45 SEC AT 72C X35], 10 MIN AT 72C |
| **Glyceraldehyde-3-phosphate dehydrogenase (*Gapdh*)** | |
| Forward: 5'-AGA GAC GGC CGC CGC ATC TTC TTG TG-3'<br>Reverse: 5'-TCT GGG TCC CAG TGA TGG CAT GG-3' | 5 MIN AT 95C, [30 SEC AT 95C, 30 SEC AT 65C,<br>45 SEC AT 72C X35], 10 MIN AT 72C |

## ***PCR primers and conditions:***

| qPCR primers | PCR Conditions: |
|---|---|
| **Insulin Receptor (*Insr*) qPCR Exon 9-10** | |
| Forward: 5'-GAGAGGCAAGCAGAGGACAG-3' | 5 MIN AT 95C, [30 SEC AT 95C, 30 SEC AT 60C, |
| Reverse: 5'-ACCAGGTCCGTGAAGGGA-3' | 30 SEC AT 72C X35], 10 MIN AT 72C |
| **Insulin Receptor (*Insr*) qPCR Exon 10-10a** | |
| Forward: 5'-TGGTGCCGAGGACAGTAGG-3' | 5 MIN AT 95C, [30 SEC AT 95C, 30 SEC AT 60C, |
| Reverse: 5'-GTGTGGTGGCTGTCACATTC-3' | 30 SEC AT 72C X35], 10 MIN AT 72C |
| **LIM domain binding protein 3 (*Ldb3*) qPCR Exon 16-17** | |
| Forward: 5'-CCTGGCATGACACCTGCTT-3' | 5 MIN AT 95C, [30 SEC AT 95C, 30 SEC AT 60C, |
| Reverse: 5'-TGCACAGTGGTTTGTCTTTCTTAG-3' | 30 SEC AT 72C X35], 10 MIN AT 72C |
| **LIM domain binding protein 3 (*Ldb3*) qPCR Exon 11-12** | |
| Forward: 5'-GCTCTGCGAAGGTCAAGCA-3' | 5 MIN AT 95C, [30 SEC AT 95C, 30 SEC AT 60C, |
| Reverse: 5'-CGGGCGACTGGGCAGAAG-3' | 30 SEC AT 72C X35], 10 MIN AT 72C |
| **Cardiac troponin T (*Tnnt2*) qPCR 3-6** | |
| Forward: 5'-GGAACAGGAAGAGCAAGAGG-3' | 5 MIN AT 95C, [30 SEC AT 95C, 30 SEC AT 65C, |
| Reverse: 5'-CAGGTTCAGCCCCACCAG-3' | 45 SEC AT 72C X35], 10 MIN AT 72C |
| **Cardiac troponin T (*Tnnt2*) qPCR Exon 7-9** | |
| Forward: 5'-GCTGAAGAAGGTCCAGTAGAGG -3' | 5 MIN AT 95C, [30 SEC AT 95C, 30 SEC AT 65C, |
| Reverse: 5'-GCACCAAGTTGGGCATGAAG-3' | 45 SEC AT 72C X35], 10 MIN AT 72C |
